# Low plasma vitamin A concentration is associated with tuberculosis in Moroccan population: a preliminary case control study

**DOI:** 10.1186/s13104-017-2737-z

**Published:** 2017-08-23

**Authors:** Mounia Qrafli, Khalid El Kari, Hassan Aguenaou, Jamal Eddine Bourkadi, Khalid Sadki, Mohammed El Mzibri

**Affiliations:** 1 Unité Mixte de Recherche Nutrition et Alimentation, (CNESTEN–Université Ibn Tofaïl), CNESTEN BP 1382 RP, 10001 Rabat, Morocco; 2 Laboratoire de Biochimie-Immunologie, Faculté des Sciences, Rabat, Morocco; 3 Service de Pneumo-Phtisiologie, Hospital Moulay Youssef, CHU Rabat, Rabat, Morocco

**Keywords:** Tuberculosis, Vitamin A, Plasma, Deficiency, Morocco

## Abstract

**Background:**

Vitamin A plays numerous roles in immune system. Its deficiency alters both the innate and adaptive immunity. Previous results reported that the micronutrients deficiency, particularly vitamin A, is observed in patients with tuberculosis. Thus, we aimed in this study to assess vitamin A concentrations in Moroccan patients with tuberculosis to set up a large efficacy study of vitamin A supplementation for TB infected patients. Plasma retinol concentration was measured by HPLC in 44 recently diagnosed TB patients and 40 healthy controls.

**Results:**

We showed that plasma vitamin A is significantly lower in tuberculosis patients as compared to healthy controls (p < 0.0001). Moreover, no significant association was found between vitamin A deficiency and, TB severity and patients’ ages.

**Conclusion:**

Our study confirms the association between low vitamin A levels and tuberculosis disease.

## Background

Tuberculosis, an infectious disease caused by *Mycobacterium tuberculosis* (*MTB*) is still a health problem causing human mortality worldwide. According to the World Health Organization (WHO) report, One-third of the world’s population is reportedly infected with *MTB* and 9.6 million new tuberculosis (TB) cases and about 1.5 million deaths were reported this year [1]. An overwhelming majority of TB patients reside in the developing countries, which suffer from marked poverty, lack of healthy living conditions, and inadequate medical facilities [2]. TB development is closely dependent on the immunological status of the hosts, requiring the presence of micronutrients including iron, zinc, vitamins A and D for adequate functioning [3, 4]. Vitamin A has a pivotal role in immune responses and accordingly is essential in the host defense against pathogens as *MTB* [5, 6]. Hall and his colleagues have reported that Th1 and Th17 immune responses are impaired in the absence of vitamin A metabolites as demonstrated by the observation of the low level of IFN-γ and IL17 cytokines production after infection of vitamin A deficient mice with *T. gondii* [7]. Indeed, the authors of this study have also demonstrated that RA restores the level of these cytokines production and therefore influences significantly the CD4 T cells protective immunity [7].

More recently, RA signaling was shown to confer Th1 cell stability and to restrain their conversion to Th17 cells [8]. In addition, it has been reported that tryptophan-aspartate containing coat protein (TACO) gene plays a crucial role allowing mycobacteria to survive within macrophages while synergistic actions of retinoic acid (RA) and vitamin D are able to down-regulate TACO transcription in human macrophages allowing delivery of Mycobacteria to lysosomes [9–11]. Furthermore, when THP-1 macrophages are exposed to chenodeoxycholic acid/RA, the entry and intracellular survival of *MTB* are significantly restricted [12]. Moreover, oral administration of RA decreases the number of colony-forming units (CFU) of *MTB* in RA-treated rats compared with controls [13].

More recently, an interesting finding revealed that expression of *NPC2* and vitamin A-mediated antimicrobial activity against *MTB* triggered a reduction in the total cellular cholesterol concentration [14].

Conversely, vitamin A deficiency (VAD) is associated with alterations in ocular tract including squamous metaplasia of the conjunctiva and cornea, in addition to keratinization and loss of cilia in the respiratory tract. Moreover, VAD is also linked to a loss of microvilli from gastrointestinal tract and a decrease in goblet cells and mucin production of genitourinary tract [15–20]. It has also been reported that VAD disrupts normal neutrophil and macrophage development and can result in impaired ability to ingest and kill bacteria [21, 22]. A high prevalence of (VAD) has been observed in patients with pulmonary tuberculosis, suggesting a strong association between vitamin A deficiency and TB development [23–25].

In Morocco, TB is considered as a major problem of the public health with a high incidence reaching, in 2014, 82 new cases for 100,000 inhabitants. Tuberculosis affects especially young adults and therefore has a high impact on the socio-economic status of the country. To fight against TB, the ministry of health has set up a national tuberculosis program focusing its interest on rapid diagnosis and adequate management of TB patients [26]. However, the national program is faced by many factors including the virulence of the bacteria, resistance to available anti-tuberculosis drugs, adherence to the treatment and the presence of malnutrition, especially micronutrients deficiency which has a dramatic impact on the immune status of patients. Therefore, modulation of the immune system of TB patients, through vitamin A supplementation, could be of a great interest to increase the rate of sputum conversion, enhance the wellbeing of TB patients and consequently help the program to fight against TB. Evaluation of vitamin A status of TB patients is primordial prior to the implementation of a nutritional strategy based on vitamin A supplementation of TB patients for better management of TB in Morocco. Thus, the present study was planned to evaluate plasma retinol concentrations in Moroccan newly diagnosed pulmonary TB patients compared to healthy controls.

## Methods

### Subjects and study design

The present study was conducted on 84 participants; 44 TB patients and 40 healthy controls. TB patients were recruited from Moulay Youssef hospital in Rabat, specialized in TB diagnosis and treatment. To be included in the study, patients have to be new TB cases and should be sputum-smear and/or culture positive. According to guidelines of the National tuberculosis program, the disease is considered as severe when it takes the form of miliary TB, multifocal TB, extended tuberculosis bronchopneumonia and in case of confection TB/HIV.

Healthy controls group was recruited from the Regional Center of Blood Transfusion of Rabat and all of them are tuberculin skin test negative. All TB patients and healthy controls recruited in this study were HIV seronegative. HIV testing was done using rapid test (Determine™ HIV-1/2 antibody test). At recruitment, each participant was assessed, using questionnaires including age, sex and medical history. Blood samples were obtained from all eligible subjects, both patients and controls, for plasma retinol evaluation.

### Blood collection

Approximately, 5 mL of the whole blood was taken by venipuncture into 5 mL vacutainers (Greiner Bio-One, Germany) containing EDTA. To avoid direct exposition to light, specimens were transported in cryoboxes. Plasma was separated after centrifugation of blood at 750*g* for 10 min at room temperature, and then stored at −80 °C until analysis.

### Plasma retinol analysis

Plasma retinol concentration was measured by HPLC (High Performance Liquid Chromatography) as previously described [27]. Briefly, 0.5 mL of serum was mixed with 0.5 mL of ethanol containing internal standard (retinyl acetate), vortexed and extracted twice with 2 mL of methylene chloride–hexane (1:5). Organic phases were pooled, evaporated to dryness and reconstituted to be injected onto the HPLC (20 μL). The retinol was measured using a Spheri-5-ODScolumn (Applied Biosystems, San Jose, CA) with mobile phase of acetonitrile–methanol (85:15). The reading was done at 326 nm using an UV–Vis detector (Model 490, Waters Associates, Milford, MA, USA). VAD was defined as plasma retinol concentration less than 0.70 mmol/L [28, 29].

### Quality control of plasma retinol analysis

The plasma retinol was measured in the laboratory of the*UnitéMixte de Recherche Nutrition et Alimentation* (*CNESTEN*–*UniversitéIbnTofaïl*), Rabat, Morocco. The precision and accuracy of the analytical method were checked periodically by the use of certified reference materials 968d (Fat-soluble Vitamins, carotenoids and cholesterol in Human Serum) from National Institute of Standard and Technology (Lot No H-479, NIST; Gaithersburg, MD, USA). The analysis of reference material showed a loss of 5% during the extractions that was considered in the calculation of serum retinol concentrations.

Otherwise, an in-house reference material (IHRM) was used (378.9 ± 3.64 µg/L). Accordingly, after 20 analysed serum samples, one IHRM sample was analysed and a Shewhart was plotted. All IHRM retinol values during the analytical period were between ±2SD. Moreover, the overall CV for the mean retinol concentration of the in-house control plasma was 0.7% within-day [Min = 0.328, Max = 1.056] and 0.988% between days.

### Statistical analysis

Differences in means between normally distributed parameters were tested using the independent student-t test. However, the retinol variable did not follow a normal distribution that’s why the median and [Min–Max] were added to the table. Mann–Whitney test was used for the comparison and differences were considered statistically significant for p values ≤0.05.

## Results

Demographic and clinical characteristics of participants are reported in Table 1. The mean age for TB patients was 31.02 ± 12 years, ranging from 18 to 52 years, whereas for healthy controls, the mean age was 38.6 ± 11.5 years, ranging from 21 to 58 years. The sex ratio (male/female) was 29/15 (1.93) and 21/19 (1.11) for TB patients and healthy controls, respectively.

**Table 1:** Demographic and clinical characteristics of TB patients and healthy controls

Variables	TB patients (N = 44)	Healthy controls (N = 40)
Age (mean ± SD)	31.02 ± 12.04	38.6 ± 11.5
Gender, number (%)		
Men	29 (59.1)	21 (52.5)
Women	15 (34.1)	19 (47.5)
HIV status	Negative	Negative
Clinical feature, number (%)		
Hemoptysis	16 (36.4)	Absence of symptoms
Expectoration	16 (36.4)	
Fever	12 (27.3)	
Cough	14 (31.8)	
Weight loss	37 (84.1)	
Abnormality shown by chest X-ray examination number (%)	37 (84.1)	

Overall, 84.1% of TB patients exhibited an abnormal chest X-ray and had a weight loss. Clinical symptoms were also reported. The most common symptoms were hemoptysis and expectoration, reported in 36.4% of patients, while chronic cough and fever were reported respectively in 31.8 and 27.3% of patients.

Plasma retinol concentration was assessed in both TB patients and healthy controls. Overall, the mean plasma retinol concentration was 1.74 ± 1.09 µmol/L in TB patients and 2.8 ± 0.97 µmol/L in healthy controls (Table 2). Comparison between TB patients and healthy controls showed a statistically significant difference (p < 0.0001).

**Table 2:** Vitamin A status of TB patients and healthy controls

Variable	All TB patients (N = 44)	Healthy controls (N = 40)	*p*
Mean retinol (µmol/L)	1.72 ± 1.09	2.8 ± 0.97	<0.0001
Median	1.40	2.55	
Minimum	0.452	1.122	
Maximum	4.945	5.394	

Distribution of plasma retinol concentration according to sex was also assessed and is reported in Table 3. No statistically significant difference was observed between men and women in both patients and healthy groups (p > 0.05). Interestingly, plasma retinol concentration was lower in TB women as compared to healthy women (p = 0.04275). Moreover, a strong statistically significant difference was recorded in TB men compared to healthy men (p < 0.0001).

**Table 3:** Distribution of plasma retinol concentration according to sex and age

	TB patients		Healthy controls		*p*
	N	Mean retinol (µmol/L) Median [Min–Max]	N	Mean retinol (µmol/L) Median [Min–Max]	
Sex					
Men	29	1.60 ± 0.8	21	2.87 ± 1.01	<0.0001
		1.45 [0.45–3.89]		2.54 [1, 39]	
Women	15	1.94 ± 1.52	19	2.69 ± 0.93	0.04275
		1.22 [0.55–4.94]		2.56 [1.12–4.73]	
		*p* > 0.05		*p* > 0.05	
Age (years)					
<29	23	1.86 ± 1.18	10	2.93 ± 0.88	<0.001
		1.45 [0.53–4.94]		2.62 [2.04–4.32]	
30–49	15	1.66 ± 1.12	19	2.98 ± 1.05	0.0014
		1.24 [0.45–4.01]		2.56 [1.17–5.39]	
>50	6	1.32 ± 0.57	11	2.30 ± 0.77	0.01
		1.24 [0.45–4.01]		2.32 [1.12–3.27]	
		*p* > 0.05		*p* > 0.05	

Distribution of plasma retinol concentration according to age was assessed in both patients and healthy groups (Table 3). Results clearly showed that plasma retinol concentration is significantly lower in patients compared to healthy controls in the three age groups. However no significant differences have been observed among the different age categories within the same group: TB patients or the healthy controls (*p* > 0.05).

According to clinical examination and chest ray interpretation, 59% of TB patients have mild TB (26/44) and 41% have severe TB (18/44). Both patients with mild and severe TB exhibited low levels of plasma retinol as compared to healthy controls. Comparison of plasma retinol concentration between patients with mild and severe TB showed no statistical difference (*p* > 0.05) (Table 4).

**Table 4:** Comparison of plasma retinol concentrations in patients with mild and severe TB

Variable	TB patients with		*p*
	Severe TB (n = 18)	Mild TB (n = 26)	
Mean retinol (µmol/L)	1.49 ± 0.86	1.87 ± 1.22	0.2614
Median [Min–Max]	1.29 [0.45–4.01]	1.40 [0.53–4.94]	

## Discussion

Vitamin A is essential for normal visual function, growth, reproduction, hematopoiesis, and immunity [18]. It can be obtained as retinyl palmitate, from dietary animal sources (liver, fish liver oils, eggs, and dairy products), and as carotenoids from vegetables (dark-green leafy vegetables and deep-orange fruits), which can be converted to retinoid within the intestine and other tissues. Metabolism of vitamin A results in the production of three major metabolites: retinol, retinaldehyde and retinoic acid (RA) [30]. RA is recognized as a principal regulatory retinoid implicated in the regulation of more than 500 genes [31], while retinaldehyde plays a unique role as the chromophore of the visual pigment rhodopsin [32, 33].

In Morocco, the vitamin A supplementation strategy is a part of a national program set up by the ministry of health, and is widely used to restore retinol status of women in post-partum and in case of severe infection, like measles. Accordingly, we have planned to evaluate the benefits of vitamin A supplementation when added to treatment of TB patients. As a first step of this evaluation, the assessment of plasma retinol in TB patients compared to healthy controls is necessary.

In this study, plasma retinol concentrations were significantly lower in TB patients than in controls, which is in phase with many studies conducted in different countries. Indeed, epidemiological evidence demonstrates an association between vitamin A and tuberculosis, and usually plasma vitamin A levels are significantly higher in healthy controls compared to tuberculosis patients [23, 24, 34–38].

It’s widely accepted that TB is the major poverty related disease and malnutrition increases susceptibility to *MTB* infection and the severity of the disease [39].

Low levels of plasma retinol observed in TB patients is well discussed and can result from a number of factors, including the decreased intake caused by the loss of appetite in TB patients [37] and the urinary loss that contributes significantly in lowering serum retinol levels in severe infections, which becomes more important in case of fever [40–42]. Moreover, plasma retinol concentration is also affected by the inflammatory status of patients. Indeed, biological evidence clearly showed that transient change in serum retinol levels is also affected by the acute phase response (APR). During the APR, the transcription and translation of positive acute-phase proteins [e.g. C-reactive protein (CRP)] are increased and positive acute phase protein concentrations correlate negatively with serum retinol concentrations [43]. In pulmonary tuberculosis, elevation of CRP is detected and a high CRP is clearly associated with more severe disease [35]. On the other hand, retinol binding protein (RBP) is the principal transport protein for delivering vitamin A from liver stores to peripheral tissues and during the APR, its production is largely decreased affecting the plasma retinol concentration [44].

Of particular interest, the use of plasma retinol evaluation as a marker in the assessment of vitamin A status is controversial. Indeed, transient changes in serum retinol that occur during the APR do not reflect changes in liver reserves and therefore, plasma retinol concentrations cannot be an adequate method to assess vitamin A status during an active APR [21, 45, 46]. Other approaches using labeled vitamin A with stable isotopes (^13^C-retinol dilution test) are widely recommended to evaluate vitamin A status giving information on plasma retinol concentration and liver stores [46].

Moreover, this study showed no significant difference between plasma retinol concentration, and the severity of TB (p = 0.2614) and the age of TB patients (p > 0.05). Few studies have explored the correlation between VAD and TB severity, among which, Pakasi and coll. have clearly shown that vitamin A was significantly lower in severe TB than in mild TB [37]. This divergence could be explained by the clinical definition of mild/severe TB and the classification criteria, as there’s no biological parameter for such stratification. Regrading the correlation of VAD with patients age, our results are in concordance with pervious reported data [47]. Interestingly, vitamin concentration is lower in TB age groups as compared to the corresponding healthy groups, and this difference is less pronounced in patients aged over 50 years, that could be due to recurrent malnutrition and decreased food intake related to elder age. In fact, recent studies reported that malnutrition and risk of malnutrition are highly prevalent among the old adults [48, 49].

Moreover, we reported here that plasma retinol concentration is lower in both men and women TB patients as compared to men and women healthy controls, respectively, and the difference is more pronounced in men. In this context, it is commonly reported that males are more vulnerable to VAD than females [18, 50–52]. On the other hand, worldwide epidemiological data reported that the majority of TB patients are males [42, 43]. Recent finding revealed that sex hormones could be a significant factor for this sex discrimination [53–55]. Indeed, testosterone is immunosuppressive and impairs the macrophages activation [56], while the estrogens are pro-inflammatory mediators able to induce the production of TNF-α and stimulate secretion of INF-γ [57, 58]. Keeping that in view, we can suggest that low plasma concentrations of vitamin A in male TB patients may rely on sex hormones, by analogy to data reported about estrogen which might regulate vitamin D_3_ metabolism [59].

## Conclusion

This study clearly showed that TB patients exhibit low concentrations of plasma retinol as compared to healthy controls and VAD could be considered as a risk factor influencing infection with *MTB*, the development and the severity of the disease. Hence, vitamin A supplementation may be a potential strategy to increase the host immunity and enhance treatment efficacy. Moreover, other approaches, such as food fortification and nutritional education, could be considered as a good strategy to prevent VAD in the whole population and therefore stimulates an effective response against TB development. Finally, further studies are required in this field to demonstrate deeply the role of Vitamin A in boosting immune system in TB patients.

## Abbreviations

*MTB*: *Mycobacterium tuberculosis*
TB: tuberculosis
RA: retinoic-acid
TACO: tryptophan-aspartate containing coat protein
VAD: vitamin A deficiency
HPLC: high performance liquid chromatography
APR: acute phase response
CRP: C-reactive protein
RBP: retinol binding protein
INF-γ: interferon-gamma
TNF-α: tumor necrosis factor-alpha
